# B Chromosomes in Genus *Sorghum* (Poaceae)

**DOI:** 10.3390/plants10030505

**Published:** 2021-03-09

**Authors:** Martina Bednářová, Miroslava Karafiátová, Eva Hřibová, Jan Bartoš

**Affiliations:** Centre of the Region Haná for Biotechnological and Agricultural Research, Institute of Experimental Botany of the Czech Academy of Sciences, Šlechtitelů 31, 779 00 Olomouc, Czech Republic; bednarova@ueb.cas.cz (M.B.); karafiatova@ueb.cas.cz (M.K.); hribova@ueb.cas.cz (E.H.)

**Keywords:** B chromosomes, supernumerary chromosomes, *Sorghum*, chromosome elimination, phylogenesis, evolution

## Abstract

B chromosomes (Bs) are supernumerary dispensable genomic elements that have been reported in several thousand eukaryotic species. Since their discovery, Bs have been subjected to countless studies aiming at the clarification of their origin, composition, and influence on the carriers. Despite these efforts, we still have very limited knowledge of the processes that led to the emergence of Bs, the mechanisms of their transmission, and the effects of Bs on the hosts. In the last decade, sophisticated molecular methods, including next-generation sequencing, have provided powerful tool to help answer some of these questions, but not many species have received much attention yet. In this review, we summarize the currently available information about Bs in the genus *Sorghum*, which has so far been on the periphery of scientific interest. We present an overview of the occurrence and characteristics of Bs in various *Sorghum* species, discuss the possible mechanisms involved in their maintenance and elimination, and outline hypotheses of the origin of Bs in this genus.

## 1. Introduction

B chromosomes (Bs) are supernumerary, dispensable chromosomes that have been observed in all major groups of living organisms—animals, plants, and fungi [1]. The basic characteristics of Bs are their inability to pair with A chromosomes (As) during meiosis and their irregular mode of inheritance [2,3]. As the transmission rate of Bs is higher than 0.5, they can be viewed as parasitic elements with their own evolutionary pathway [4]. One of the common features of Bs is that they are present only in some individuals of a particular species, and this variability may also exist at the level of populations or even at the level of tissues of a single individual. Most Bs share the common basic features mentioned above, but they have also developed some species-specific attributes, which in some cases resulted in the emergence of unique systems of Bs [5].

Bs are known to be present in a number of plant species, however, they have been studied in more detail mainly in plants with agronomic importance (e.g., rye, maize) [6,7,8,9,10,11,12,13]. Information about Bs in other plant species is rather superficial. In angiosperms, Bs tend to be present in species that have relatively large genomes and a small number of chromosomes [14,15,16]. Bs were found in 8% monocots and 3% eudicots, and their distribution in various orders, families, and genera is not random [17]. Among monocotyledonous plants, the orders Commelinales and Liliales seem to be the “hotspots” of B chromosome occurrence [17].

Based on the dispensable nature of Bs and on their potentially detrimental effect on a host, it would be logical to expect that they will be gradually suppressed and subsequently eradicated from the population. However, Bs seem to successfully persist in the populations thanks to their specific accumulation mechanisms [18]. One of these mechanisms is nondisjunction, which has been relatively well described in rye and maize [12,19,20,21,22]. In maize, nondisjunction takes place during the second pollen mitosis, when two sperm cells are formed. Sister chromatids of B chromosome fail to disjoin at anaphase; both are pulled to one pole and thus end up in one sperm cell. As a result, one of the sperm cells accumulates B chromosome at the expense of the other. The sperm cell with B chromosome then preferentially merges with the egg cell [20,23,24,25]. In rye, nondisjunction occurs during the first pollen mitosis, when vegetative and generative nuclei are formed: both chromatids of the B chromosome are in most cases included in the generative nucleus [26]. Nondisjunction of rye and maize Bs are both examples of post-meiotic drive. Besides this mechanism, pre-meiotic and meiotic drive have also been reported, but mainly in animals [27,28,29,30].

Generally, Bs are smaller than As, but Bs of similar size as As (“large” Bs) have also been reported [2,31,32]. In many species, different morphological variants of Bs have been observed within a single species, for example, in chives *Allium schoenoprasum*, grasshopper *Eyprepocnemis plorans*, or fish *Astyanax scabripinnis* [33,34,35]. Bs occur in different organisms at variable numbers, and the tolerable maximum depends on the particular species. When present in low numbers, Bs generally do not have any detrimental effect on the host, however, in higher numbers they can reduce the fitness of the carrier [2]. There have been a few reports suggesting some positive effects of the presence of Bs on their carrier [36,37,38], but in general, Bs do not provide any obvious benefits.

Due to the absence of selection pressure, Bs behave like a “genomic sponge” and accumulate sequences of various origins. As shown in rye and maize, Bs can accumulate organellar DNA, transposable elements, satellite sequences, ribosomal DNA, and other sequences from various As [39,40,41]. Bs can also contain genic sequences, but they are mostly not functional due to the pseudogenization [42,43,44]. All captured sequences can then diverge from their original homologues, and thus Bs can serve as a potential source of genetic variability. Although Bs have been considered transcriptionally inactive elements, recent studies indicate that at least some Bs are transcriptionally active and contain functional protein-coding genes [45,46,47,48]. Genes localized on Bs were shown to play a role in female sex determination in cichlid fish [49], in the processes related to cell division in *E. plorans* [48], and in the cell cycle and development in the red fox (*Vulpes vulpes*) and raccoon dog (*Nyctereutes procyonoides procyonoides*) [50]. These data suggest that Bs can carry genes controlling their specific behavior.

## 2. B Chromosomes in the Genus *Sorghum*

**Figure 1:**
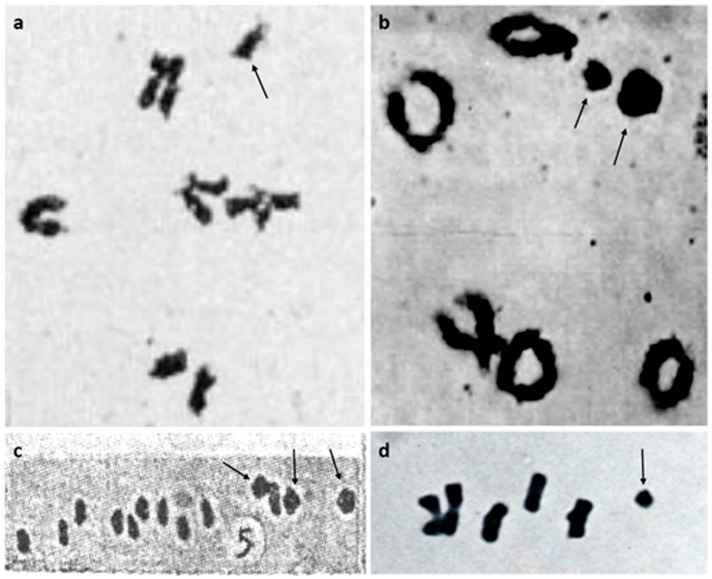
Chromosome pairing in meiotic metaphase I in pollen mother cells of *Sorghum* sp. Bs are marked by arrows. (**a**) Five A-bivalents and one B-univalent of *S. stipoideum* [52] (reprinted with permission from Springer Nature: Nature, Heredity, *B-chromosomes in Sorghum stipoideum, Wu*, Copyright 1992); (**b**) Five A-bivalents and two B-univalents in *S. purpureosericeum* [54] (copied from *Morbid mitosis and the activity of inert chromosomes in Sorghum*, Darlington and Thomas (1941) with the permission of the publisher); (**c**) Ten A-bivalents and three B-bivalents of *S. halepense* [56] (modified from *Paternal transmission of accessory chromosomes in a species of Eu-sorghum*, Raman et al. (1965)); (**d**) Five A-bivalents and one B-univalent of *S. nitidum* [57] (copied from *Accessory chromosome in Sorghum nitidum Pers*., Wu and Pi (1975) with the permission of the publisher).

In the genus *Sorghum*, Bs have been reported in five species: *S. bicolor* ssp. *verticilliflorum* [51], *S. stipoideum* (Figure 1a) [52], *S. purpureosericeum* (Figure 1b) [53,54], *S. halepense* (Figure 1c) [55,56], and *S. nitidum* (Figure 1d) [31,57]. Despite the morphological variability of Bs described in sorghums, they share one common feature—they are well preserved in the cell lineages leading to the reproductive organs, but are absent in most somatic tissues. Several cytological studies on *Sorghum* Bs have been performed, and their morphology and behavior during meiosis have been relatively well documented in all species except *S. bicolor* ssp. *verticilliflorum*.

From all the Bs in genus *Sorghum*, the most detailed information is available about Bs from *S. purpureosericeum*. A maximum of six Bs in one cell was reported in this species [53], and the Bs described so far are not morphologically identical. Darlington and Thomas [54] described three types of heterochromatic Bs (long, medium, short), which did not pair with each other. Based on the published reports, the medium type of B chromosome with visible constriction seems to be the most common. The transmission of B chromosome(s) through meiosis in both 1B and 2B plants has been well documented, showing nearly regular behavior in 2B plants, resulting in four microspores with 1B chromosome. In 1B plants, however, the B chromosome passes undivided through the first meiotic division and divides in a second division, giving rise to two microspores with 1B and two without [58]. Meiosis of B-carrying plants was also previously studied by D’cruz and Deshmukh [59], who found precociously dividing B chromosome at metaphase I of male meiosis. An outline of the B chromosome behavior in the first pollen mitosis was proposed by Darlington and Thomas [54], who also suggested a unique manner of the accumulation of Bs in this species. They observed extra divisions (polymitosis) between the first and second pollen mitosis, which they believed led to B chromosome multiplication [54]. The existence of the micronucleus containing Bs in resting cells has also been noticed, and the theory of B chromosome elimination via micronucleation has been previously proposed in earlier reports [52,54,58].

In *S. nitidum*, Raman and Krishnaswami [31] observed Bs in diploid plants (2n = 2x = 10), but not in tetraploids (2n = 4x = 20). The size of Bs was equal to the chromosomes of A complement. When two Bs were present, they paired regularly and behaved normally at meiosis. Wu and Pi [57], and later Wu [60], analyzed *S. nitidum* plants with one B chromosome (2n = 2x = 10 + 1B). They described B chromosome as an isochromosome, which folded back to pair with itself at the pachytene. The whole chromosome was heterochromatic with a terminal knob distal to the centromere, and its heterochromatic arm was separated from the knob by a constriction. This B chromosome was much shorter than any chromosome of the A-complement, which indicates that *S. nitidum* might contain more than one type of B chromosome.

The only study on the Bs in *S. stipoideum* was published by Wu [52]. The author found one type of B chromosome, which was distinctly shorter than any of the As and euchromatic along its whole length. Its euchromatic nature is interesting in the context of the fact that the other Bs in the *Sorghum* genus are heterochromatic, and it implies transcriptional activity. However, the euchromatic nature of Bs is not striking, as, for instance, *Allium cernuum* and *Crepis pannonica* also carry partially or completely euchromatic Bs [61,62]. The B chromosome of *S. stipoideum* was described as an isochromosome, which exhibited inter-arm pairing when present only in one copy. At anaphase I of 1B plants, B chromosome divided precociously or moved undivided to one pole of the cell. During anaphase II, the majority of the cells had lagging B-chromatids and after division, micronuclei were observed, indicating B chromosome elimination. In 2B plants, meiotic behavior was nearly regular. However, numerical variability among and even within the spikelets was observed [52].

In *S. halepense*, four to six Bs have been observed, and their occurrence seems to be limited exclusively to diploids (2n = 2x = 20) [63]. The behavior of Bs during meiosis in pollen mother cells was aberrant; accessory bivalents exhibited delayed disjunction or nondisjunction leading to the subsequent elimination. Three types of Bs were reported, two of which showed partial homology [56,63].

The presence of B chromosome in *S. bicolor* ssp. *verticilliflorum* is rather questionable. Huskins and Smith [51] observed an additional pair of chromosome fragments during male meiosis in this species. These fragments were much smaller than As and were attached to a bivalent of As. They considered those fragments to be a pair of supernumerary chromosomes. However, as this study is the only existing work describing the presence of Bs in this species, it is questionable whether it was a real B chromosome or rather a mere chromosomal fragment. It has been documented that some chromosomes contain so-called “fragile sites” that are prone to breakage during cell division and are sensitive to replication stress [64]. In plants, these sites are associated with 45S rDNA. Fragile sites can lead to chromosomal rearrangements and affect genome organization [65,66,67]. In the case of *S. bicolor* ssp. *verticilliflorum*, fragile sites in some genotypes might lead to chromosomal fragments misinterpreted as Bs. Unfortunately, so far there has been no other research that could confirm or disprove the existence of Bs in this species.

## 3. Elimination and Maintenance of B Chromosomes

Although Bs are usually transmitted regularly in mitosis, sorghums belong to species where the transmission is irregular. Bs in genus *Sorghum* show a high level of numerical instability, which is frequently observed in somatic tissues. In all *Sorghum* species, B chromosome elimination or irregular transmission leads to a mosaic distribution of the Bs. In *S. purpureosericeum*, Janaki-Amal [53] reported B chromosome absence in roots. Darlington and Thomas [54] described B chromosome absence in root, stem, and leaf tissues in the same species. These findings correspond with the results of a recently published study [68], in which the authors analyzed parts of adult B-carrying plants in order to identify the tissues where the B chromosome is preserved. Except for the inflorescence, where Bs are stably present, the residual population of B-carrying nuclei was detected in leaf meristem, last node, and peduncle. Thus, in *S. purpureosericeum*, Bs probably persist only in the meristems from which generative organs are later established, and are likely to be eliminated from other vegetative tissues [68]. Similarly, B elimination from root tissue was noticed in *S. nitidum* [31] and *S. halepense* [56]. In microsporocytes and tapetal cells of *S. stipoideum*, Bs occurred mosaically, while root, stem, and leaf meristem cells were completely lacking Bs [52]. Recently, the process responsible for the elimination of B chromosome from the roots of *Aegilops speltoides* has been described [69]. The strictly controlled process is based on B chromatid nondisjunction in mitosis, lagging in anaphase, and the formation of a micronucleus, which is subsequently eliminated. Elimination mechanisms in *Sorghum* have not yet been investigated, but they might be similar to *Aegilops*.

Research on other B-containing species indicates that the existence of accumulating mechanisms is necessary to avoid the loss of Bs [70]. The Bs in genus *Sorghum* also undoubtedly had to evolve some accumulation mechanism(s) acting directly against natural selection. However, our knowledge of these multiplicative mechanisms is only fragmentary. If we consider possible divisions where the nondisjunction can take place, the meiotic drive can be ruled out, as the division of pollen mother cells was proven to be regular [54,58]. As pre-meiotic drive is generally rare, attention should be focused mainly on pollen mitosis. A solitary study of Darlington and Thomas [54] is the only work dealing with the division following male meiosis. The authors did not find any irregularities in first pollen mitosis and suggested that nondisjunction occurs during second pollen division. Conclusions presented in this work were drawn from the statistical analysis of progenies of B-carrying plants, and the study lacks strong proof of this hypothesis. This approach, based on the analysis of the frequency and number of Bs in the offspring, can be replaced today by technologically advanced methods, which enable the visualization of the B chromosome in situ, directly on its way through both pollen mitoses. These modern approaches have already been used to elucidate the mechanism of nondisjunction in rye or *Aegilops* [12,71]. Markers recently developed for *S. purpureosericeum* [68] open up the possibility of also using these approaches in *Sorghum*.

**Figure 2:**
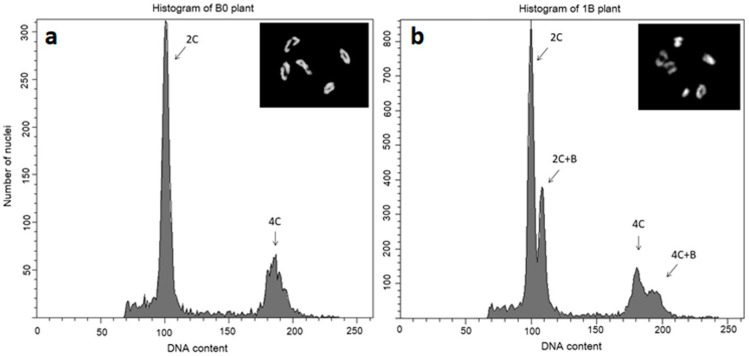
Flow cytometric analysis of the nuclei of *Sorghum purpureosericeum* isolated from spikelets. The cytological verification of B chromosome presence/absence in the analyzed plant is shown in the inset. (**a**) Histogram of B-negative plant showing two distinct peaks corresponding to 2C and 4C nuclei; (**b**) histogram of B-positive plant with a significant change in the flow karyotype. Even one copy of the B chromosome results in a clear separation of the populations of nuclei carrying B chromosome at both 2C and 4C ploidies.

The tissue-specific elimination of Bs complicates their detection in growing plants. In the sorghum model, the detection of Bs requires the cultivation of the plant up to the stage of inflorescence, when immature anthers are collected and the meiocytes are scored at metaphase I, when the presence of Bs can be determined. In species with a specific proportion of A and B chromosomes, an alternative approach based on flow cytometry can be used (Figure 2) [68]. Although the flow cytometry screening method also requires the inflorescence, it is less laborious and thus facilitates and speeds up the whole detection process. The protocol was originally established for *S. purpureosericeum* and worked well both for isolated haploid nuclei from pollen grains and for samples prepared from whole florets. However, this approach is not suitable for the detection of Bs in very young seedling/seeds, as it requires a relatively large amount of material and thus is destructive. The flow cytometry approach has previously been used to detect Bs in leaves and immature embryos of *Aegilops speltoides* [69,71]. The evaluation of B status in developing seeds would make the work significantly faster, however, unfortunately, this kind of approach based on PCR markers is not currently available.

Cytological techniques play an irreplaceable role in B chromosome research, but only molecular studies are able to provide us with information that is above the resolution of cytogenetics. Sequencing is a powerful tool that can bring us information about the genomic content, origin, evolution, and biological role of Bs. Next-generation sequencing, enabling large-scale analysis, provides an opportunity for a significant advance in B chromosome research. However, out of the five types of Bs reported in *Sorghum*, only the B chromosome in *S. purpureosericeum* has been subjected to molecular studies so far [68]. Sequence analysis has revealed several B-specific repeats in this chromosome, including DNA transposon/hAT and one LINE element. Based on the selected repetitive sequences, PCR and cytogenetic markers specific for B chromosome have been developed [68]. The accumulation of different types of repeats in Bs is common; these repeats are often strongly amplified and may even form a significant part of the B chromosome, like the PSR element in *Nasonia vitripennis* [72] or micro B of *Brachycome dichromosomatica* [73]. B-specific repeats have also been identified in other plant species, such as E3900 and D1100 in rye [10,13], ZmBs and StarkB in maize [7,74], and Bd49 in *B. dichromosomatica* [75].

## 4. Did B Chromosomes Emerge Several Times in the Genus *Sorghum*?

**Figure 3:**
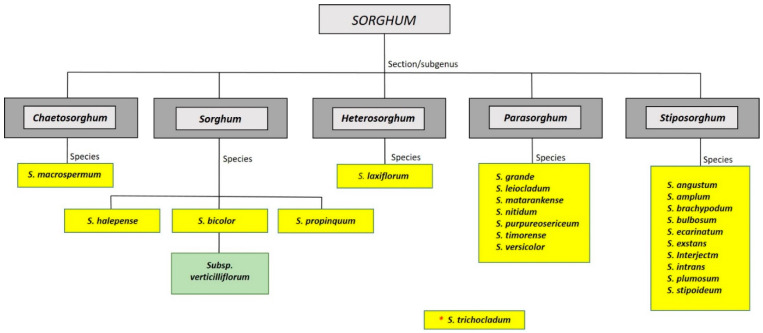
Classification of *Sorghum* (modified from Ananda et al. [78]) * *S. trichocladum* has not yet been assigned to any subgenus.

Sorghum is a genus of monocot flowering plants in the grass family Poaceae, subfamily Panicoideae, and the tribe Andropogoneae. The genus includes 23 annual and perennial species and a number of subspecies and races resulting from hybridization. Based on morphological traits, they are divided into five subgenera: *Sorghum*, *Parasorghum*, *Stiposorghum*, *Chaetosorghum*, and *Heterosorghum* (Figure 3) [76]. Subgenus *Sorghum* is represented by cultivated sorghum (*Sorghum bicolor* (L.) Moench) and its wild relatives. Representatives of this subgenus originated in Africa and Asia, and their chromosome numbers are 2n = 2x = 20 in diploids and 2n = 4x = 40 in tetraploids [77]. The subgenus *Parasorghum* includes seven species from Australia, Central America, Africa, and Asia [77], and their chromosome numbers vary from 2n = 2x = 10, 20, 30 to 40. *S. macrospermum* (2n = 2x = 40) is the only representative of the subgenus *Chaetosorghum* and can be found endemically in the Northern Territory of Australia. Subgenus *Heterosorghum* is represented by *S. laxiflorum*, growing in Northern Australia and Papua New Guinea (2n = 2x = 40). The last subgenus, *Stiposorghum*, includes 10 species occurring in Northern Australia with chromosome numbers ranging from 2n = 2x = 10, 20, 30 to 40 [77].

**Figure 4:**
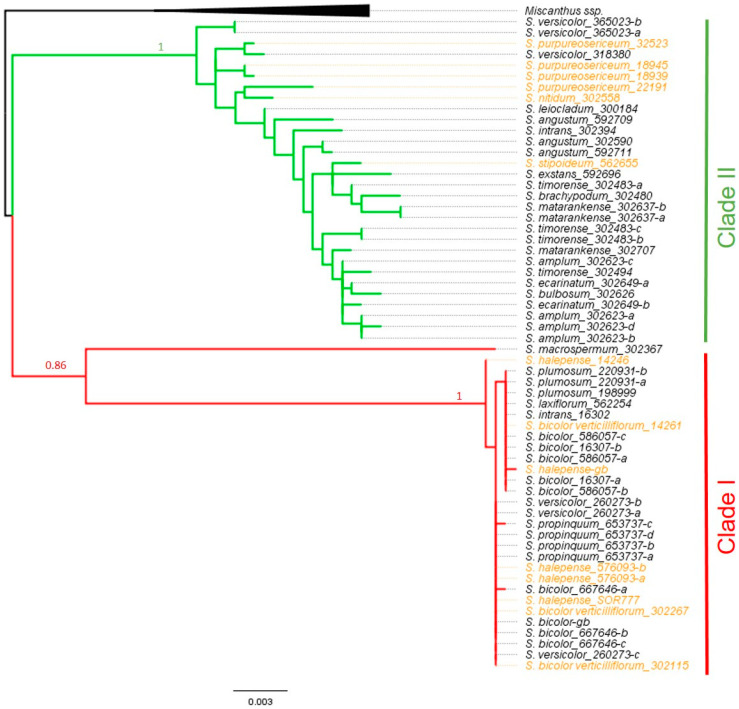
Phylogenetic tree of 21 *Sorghum* species based on phyML analysis using concatenated nuclear and chloroplastic sequences (ITS1-ITS2-trnHpsbA-trnLtrnF). With strong branch support, two main clades I and II are resolved. B-carrying species are marked in orange.

The genus *Sorghum* has been subjected to several phylogenetic analyses [79,80,81,82], most of which agree with this classification of species into the abovementioned sections, although relationships between some sister taxa are still under debate. For example, a close relationship between *S. macrospermum* and *S. laxiflorum* led to proposals to merge the *Chaetosorghum* and *Heterosorghum* sections [80,81,82]. We have performed phylogenetic reconstruction of the genus with a focus on species possessing Bs. PhyML analysis of concatenated sequences of ITS1-ITS2, trnH-psbA, and trnL-trnF resulted in a phylogram in which two strongly supported major clades I and II were identified (Figure 4), which is in agreement with the phylogenetic analyses published previously.

A widely accepted hypothesis of the formation of Bs assumes that they have autosomal origin. This is supported by the fact that sequences similar to those from As have often been found on Bs. Bs containing mosaically organized sequences derived from different As have been described, for example, in rye [39], maize [83], and *Brachycome dichromosomatica* [73]. In *Nasonia vitripennis*, B chromosome was formed from interspecies hybridization [84]. Additionally, Bs originating from sex chromosomes were described in grasshopper *Eyprepocnemis plorans* and frog *Leiopelma hochstetteri* [85,86]. Despite countless studies dealing with the possible origin and evolution of B chromosome(s) [7,39,84,87,88,89,90], these issues still remain unclear.

**Figure 5:**
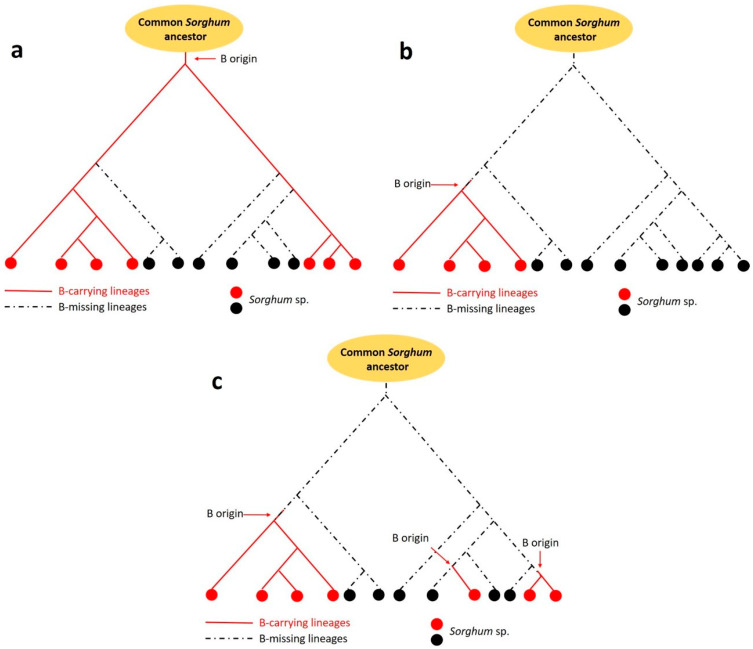
Schemes of the possible origin of B chromosome in the genus *Sorghum*. (**a**) Origin of B chromosome(s) in a common ancestor; (**b**) origin of B chromosome(s) in a group of closely related species; (**c**) multiple origins of B chromosome(s).

There are three possible evolutionary scenarios for B chromosome(s) in the genus *Sorghum* (Figure 5). B chromosome(s) may have formed (1) in a single event in a common ancestor of all *Sorghum* species and then have been preserved in some lines during evolution and disappeared in others (Figure 5a); (2) once in the ancestor of closely related species, all of which have kept Bs up until today (Figure 5b); or (3) several times during independent events in *Sorghum* evolution, which includes also the possibility that the B chromosome originated only at the level of individual species (Figure 5c).

Considering the conclusions of the cytological studies [31,51,52,53,54,55,56,57,58,59,60,63], it is clear that there are several types of Bs within the genus, which differ in their morphology. One evolutionary scenario assumes the formation of the B chromosome in a single event and its persistence in closely related species. Given that Bs occur in both clades (Figure 4), we can exclude a close phylogenetic relationship between the species carrying Bs. However, the phylogenetic analysis cannot rule out the possibility that B chromosome originated only once during the early evolution of *Sorghum* or in the ancestor of the genus. This hypothesis could be supported or questioned based on the analysis of sequence similarity between the Bs from different species in the genus *Sorghum*, however, these data are not currently available. Recently, Wu et al. [71] identified B-specific tandem repeat shared by Bs in *Aegilops speltoides*, *Aegilops mutica*, and *Secale cereale*, however, they were not able to conclude whether the chromosomes have a common origin or whether the shared repetitive sequence is a result of the exchange of genetic material among those species.

The hypothesis assuming multiple independent origins of Bs in the genus *Sorghum* is also feasible. This scenario of B chromosome origin appears to be supported by the nature of the B chromosome of *S. nitidum*—the appearance of B in this species might represent an example of the formation of the B chromosome at the species level, as has already been hypothesized by Wu and Pi [57] and Wu [60]. The B chromosome in *S. nitidum* is an isochromosome, and its arms strikingly resemble the short arm of the nucleolus-associated chromosome, which is also entirely heterochromatic and approximately similar in length. Since B chromosome does not pair with the nucleolus-associated chromosome, further structural and genic changes had to occur later, leading to the loss of homology and to the inability of B chromosome to pair with the short arm of the nucleolus-associated chromosome [60]. Such resemblance between Bs and As has not been found in any other *Sorghum* species. However, since Bs are expected to be prone to aberrations and the accumulation of mutations [11,91], a relatively rapid diversification and loss of ability to pair with the original A homologue can be assumed. It is possible that the B chromosome of *S. nitidum* is evolutionarily younger than other *Sorghum* Bs, and therefore a high level of similarity between A and B is still maintained in this species.

Another argument speaking for the independent origin of Bs in different *Sorghum* species is the possibility of intra- and inter-specific hybridization. It has been suggested that if hybridization occurs between separated, diverged subpopulations, various irregularities in meiosis may appear, which is a precondition for the B chromosome formation [60]. Namely, the subgenus *Sorghum* is a complex group that includes a number of closely related species, subspecies, and races that can interbreed freely, and some species (e.g., *S. halepense*) are assumed to have hybrid origin [92,93,94]. Frequent hybridization might represent conditions favorable for the formation of Bs. The origin of Bs through interspecific hybridization has been demonstrated in hybrid derivatives from spontaneous crossing between two *Coix* species [95] and has also been described in *Poecilia formosa* and *Nasonia vitripennis* [84,96,97].

## 5. Conclusions

B chromosomes (Bs) are unique genomic elements with a transmission rate higher than 0.5. Although our knowledge of Bs has advanced considerably since their discovery in the first half of the last century, many questions remain unanswered. The elusiveness of *Sorghum* Bs, resulting from their extensive elimination during early plant development, certainly contributed to the fact that they have not yet been subjected to any comprehensive research. In the last century, several authors have provided cytological characteristics of Bs in some *Sorghum* species, but since then the research in this field has hardly progressed. However, new technologies give us new opportunities to meet this challenge. The use of flow cytometric screening and sorting makes it easier to detect the presence of Bs and to obtain material for sequencing, which will be necessary for their thorough molecular characterization. So far, our knowledge of the mechanisms of accumulation or somatic elimination of Bs in *Sorghum* is only marginal. Analysis of the effects of *Sorghum* Bs on gene expression is another interesting topic that deserves thorough investigation. Research on Bs in *Sorghum* is still in its infancy, and there is a long way to go before we discover at least some of the mechanisms behind the unique behavior of these enigmatic elements in this genus.

## Data Availability

Not applicable.
